# Adolescents’ trajectories of depression and anxiety symptoms prior to and during the COVID-19 pandemic and their association with healthy sleep patterns

**DOI:** 10.1038/s41598-024-60974-y

**Published:** 2024-05-10

**Authors:** Serena Bauducco, Lauren A. Gardner, Scarlett Smout, Katrina E. Champion, Cath Chapman, Amanda Gamble, Maree Teesson, Michael Gradisar, Nicola C. Newton

**Affiliations:** 1 Flinders University, Adelaide, Australia; 2 Center for Health and Medical Psychology, Örebro University, Fakultetsgatan 1, 701 82 Örebro, Sweden; 3 The Matilda Centre for Research in Mental Health and Substance Use, The University of Sydney, Sydney, Australia; 4 The Woolcock Institute, Macquarie University, Sydney, Australia; 5 WINK Sleep Pty Ltd, Adelaide, Australia; 6 Sleep Cycle AB, Gothenburg, Sweden

**Keywords:** Risk factors, Depression, Anxiety

## Abstract

The COVID-19 pandemic has seen a rise in anxiety and depression among adolescents. This study aimed to investigate the longitudinal associations between sleep and mental health among a large sample of Australian adolescents and examine whether healthy sleep patterns were protective of mental health in the context of the COVID-19 pandemic. We used three waves of longitudinal control group data from the Health4Life cluster-randomized trial (N = 2781, baseline M_age_ = 12.6, SD =  0.51; 47% boys and 1.4% ‘prefer not to say’). Latent class growth analyses across the 2 years period identified four trajectories of depressive symptoms: *low-stable* (64.3%), *average-increasing* (19.2%), *high-decreasing* (7.1%), *moderate-increasing* (9.4%), and three anxiety symptom trajectories: *low-stable* (74.8%), *average-increasing* (11.6%), *high-decreasing* (13.6%). We compared the trajectories on sociodemographic and sleep characteristics. Adolescents in low-risk trajectories were more likely to be boys and to report shorter sleep latency and wake after sleep onset, longer sleep duration, less sleepiness, and earlier chronotype. Where mental health improved or worsened, sleep patterns changed in the same direction. The subgroups analyses uncovered two important findings: (1) the majority of adolescents in the sample maintained good mental health and sleep habits (*low-stable* trajectories), (2) adolescents with worsening mental health also reported worsening sleep patterns and vice versa in the improving mental health trajectories. These distinct patterns of sleep and mental health would not be seen using mean-centred statistical approaches.

## Introduction

Mental disorders are the leading cause of disability among young people aged up to 24 years worldwide, accounting for one quarter of all years lived with disability^1^. In Australia, mental disorders make up three of the five leading causes of burden of disease among those aged 12–24 years^2^ and cost the economy approximately $200–220 billion yearly^3^. The COVID-19 pandemic has caused disruptions to all facets of adolescent development and exacerbated mental health issues such as depression, anxiety and self harm^4–7^. Examining modifiable factors that were protective of mental health throughout this global crisis can offer insights on targets for prevention and early intervention, especially since improving the mental health of young people is a critical public health priority^8^.

There is growing evidence that sleep is a key modifiable factor associated with mental health^9–14^. Human sleep is primarily under biological control, governed by the well-supported two-process model of sleep^15,16^. The first process is sleep homeostatis, whereby pressure to sleep builds across the waking day and evening and dissipates during sleep. As adolescents develop, their ability to build sleep pressure in the evening declines, resulting in increased alertness in the evening. This inevitably results in a delay in the onset of sleep (i.e., a longer sleep onset latency)^16^. The second process—the circadian rhythm—is a cycle of sleep and wake across the 24 h day, and is of particular relevance during adolescence. From about age 10 to 20 years, the timing of the circadian rhythm delays, resulting in a later onset of sleep in the evening, and a later rise time (when allowed to sleep-in)^16^. However, 5 out of 7 days of the week, adolescents are prevented from sleeping at their natural circadian times as they need to wake relatively early to prepare for, travel to, and attend school^17^. This inevitably results in a restriction of sleep across the school week (i.e., shortened total sleep time) and weekend catch-up, which leads to irregular sleep and pepetuates weekday sleep debt^17^.

Multiple studies have shown that poor sleep during paediatric development can increase the odds of developing anxiety and depression, with two mechanisms implicated^9^. First, the restriction of sleep (less total sleep time) has been shown to dampen positive mood and hinder one’s next-day emotion regulation^9^. Second, an extended sleep onset latency is proposed to create an ideal environment for repetitive negative thinking (i.e., worry and rumination), which has been linked to both depression and anxiety in young people. An extended sleep onset latency is common among later chronotypes, which may be a predisposing factor for the development of depression and anxiety symptoms^18^. Conversely, a healthy sleep profile (particularly short sleep onset latency—between 5 and 30 min^19^, sufficient total sleep time—between 8 and 10 h^20^, and less daytime sleepiness) is associated with increased mental resilience (i.e., lower symptoms of anxiety and depression)^21,22^. Therefore, sleep is an important risk-/protective factor for the development of mental ill-health.

During the COVID-19 pandemic, mental resilience was tested on a global scale, and many adolescents experienced heightened psychopathology^4–7^. Sleep was also impacted, potentially relating to heightened stress and restrictions on movement and closure of schools; however, evidence as to whether healthy sleep improved or worsened has been mixed^23–25^. Surprisingly, one longitudinal study found that the prevalence of depressive symptoms, anxiety symptoms and sleep problems was higher before, compared to during and after, the pandemic^26^. While studies focusing on average changes are informative, they might miss *for whom* changes occur. Did all adolescents experience worse sleep and mental health? Were adolescents with healthy sleep patterns more resilient? For example, later chronotype^26,27^, shorter sleep duration^27^ and insomnia symptoms^28^ were associated with higher risk for adolescents’ mental ill-health during the pandemic. Identifying subgroups of adolescents at lower risk for mental ill-health during the pandemic, and thoroughly examining protective sleep characteristics, can inform key targets for prevention and early interventions in this population. Previous studies, however, have examined changes across their entire samples, and have examined only a few interindividual differences in sleep patterns.

To address this gap, we utilised data from pre-pandemic (2019), and during the pandemic (in the latter half of 2020 and 2021) among a large sample of early- to mid-Australian adolescents^29^. Using a person-centred analysis, we examined whether adolescents who experienced lower and stable levels of anxiety and depression before and during the pandemic had healthier sleep patterns (i.e., shorter sleep onset latency and night-time awakenings, sufficient total sleep time, low levels of sleepiness, an earlier chronotype, and shorter catch-up sleep on weekends) compared to adolescents experiencing poor and worsening mental health symptoms.

## Results

### Descriptive statistics

The analytical sample of the current study consisted of 2781 students (baseline Mage = 12.6, SD = 0.5; 47% boys and 1.4% ‘prefer not to say’). The largest group was from New South Wales (49.8%), followed by Western Australia (28.1%), and Queensland (22.2%). The average perceived socioeconomic status in the sample was 9.43 (range 0–13; SD = 1.85). The majority of adolescents were born in Australia (86.2%) or another English-speaking country (6.9%). In line with this, the majority reported speaking English at home (93.1%). Means and standard deviations of depressive and anxiety symptoms and sleep variables (sleepiness, weekday sleep duration, sleep onset latency [SOL], wake after sleep onset [WASO], and chronotype) are presented in Table 1.

**Table 1:** Means and standard deviations among the study variables over time.

		T1 (2019)	T2 (2020)	T3 (2021)
Mental health variables Mean (SD)	Depressive symptoms	3.39 (3.83)	4.13 (4.58)	4.65 (4.83)
	Anxiety symptoms	23.25 (10.61)	24.51 (12.22)	25.34 (12.74)
Sleep variables Mean (SD)	SOL (min)	41.0 (50.0)	35.2 (45.5)	35.6 (50.7)
	WASO (min)	19.6 (37.6)	14.3 (32.6)	12.6 (30.0)
	TST (h)	8.10 (1.8)	7.89 (1.6)	7.67 (1.6)
	Sleepiness	13.80 (6.14)	14.27 (6.34)	15.30 (6.21)
	Chronotype	3.3 (1.2)	3.7 (1.2)	4.0 (1.3)
	Sleep-in_we_ (min)	88.6 (82.6)	93.9 (80.54)	107.9 (85.7)

Age range (11–14), Depression scores range (0–18), anxiety scores range (13–65), sleepiness scores range (0–32).

*SOL* sleep onset latency, *WASO* wake after sleep onset, *TST* total sleep time, Chronotype = Midpoint of Sleep on Free-days (corrected for weekday sleep), Sleep-in_we_ = weekend wake-up time–weekday wake-up time. All sleep measures refer to weekdays, except for Chronotype and Sleep-in_we_.

### Changes in depressive and anxiety symptoms over time

Two separate latent growth curve models were estimated to examine whether and how adolescents’ depressive and anxiety symptoms changed over time, from early to mid-adolescence and throughout the pandemic. Both yielded a good model fit (see Table 2). The mean of the slope was positive and statistically significant for both depression and anxiety symptoms, which indicates that on average, participants reported increasing symptoms over time. The variances of the slope and the intercept were both statistically significant, which indicates that adolescents differed both in the level of depressive and anxiety symptoms at T1 and in how they changed from T1 to T3 (see Table 2). Therefore, we went on to explore subgroups of adolescents following different trajectories of depressive and anxiety symptoms.

**Table 2:** Model fit, intercept and growth estimates for the linear change model in depression and anxiety symptoms over 2 years.

	Model fit statistics							Intercept		Slope	
	Chi-sqr (df)	*p*	CFI	RMSEA	90% CI	*p*	SRMR	Mean	Variance	Mean	Variance
Depression	48.03 (3)	< 0.001	0.96	0.073	0.056, 0.092	0.014	0.054	3.42***	5.70***	0.70***	1.44***
Anxiety	41.17 (3)	< 0.001	0.97	0.068	0.050, 0.087	0.048	0.06	23.32***	57.26***	1.18***	10.77***

N = 2781, Estimator = MLR. *p < 0.05, **p < 0.01, ***p < 0.001.

### Depressive symptoms subgroup trajectories

**Figure 1:**
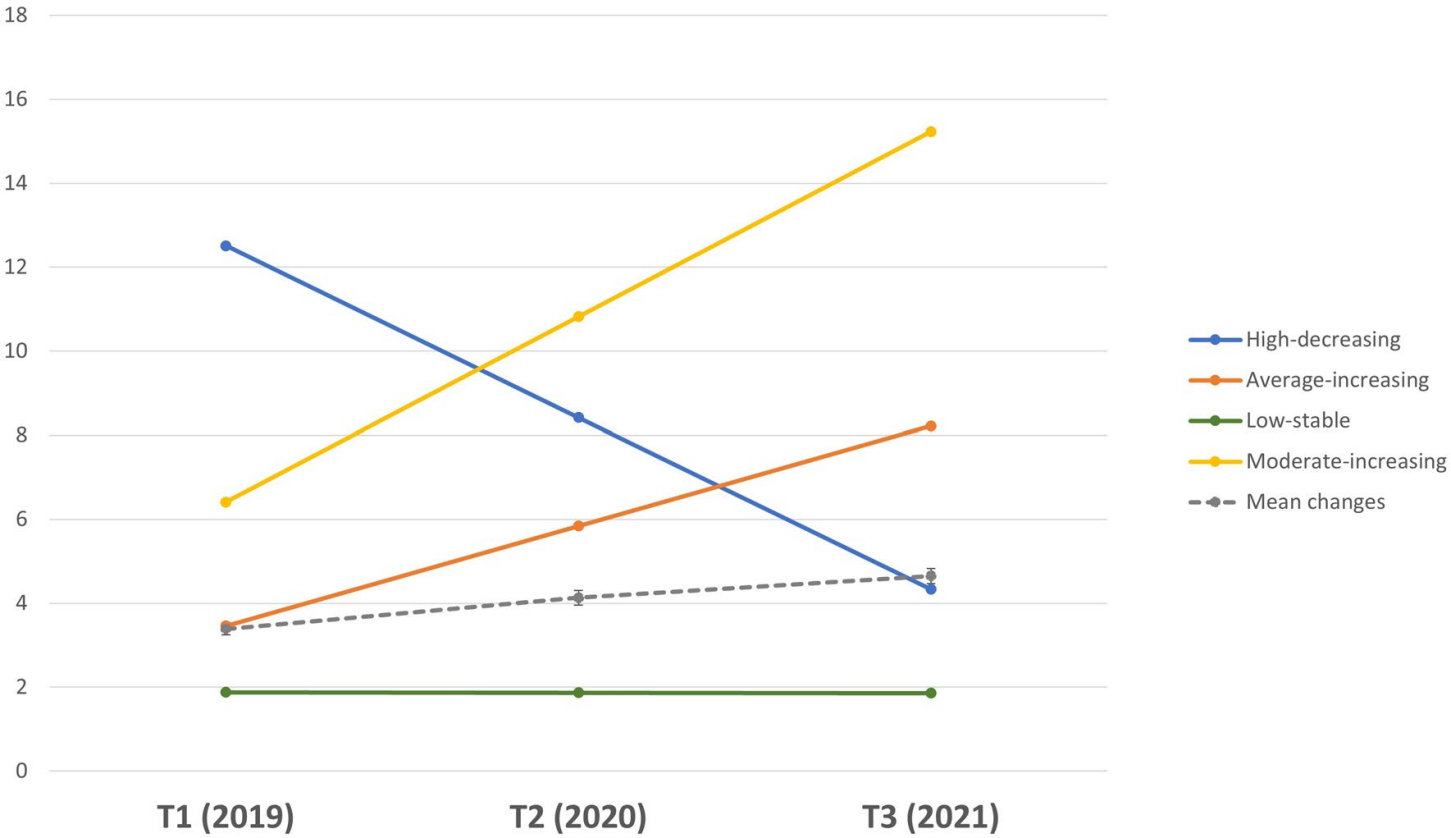
Trajectories of depressive symptoms throughout the pandemic. *Note*. The dashed line is the average score for the whole sample at T1-T2-T3. Depressive symptom scores ranged from 0–18.

We used latent class growth analysis (LCGA) to identify subgroups of adolescents who followed different trajectories of depressive symptoms over time and found four different trajectories (see “Supplementary material”). The first class (*high-decreasing*) included 7.1% of the sample. Adolescents in this class reported high levels of depressive symptoms at T1 and average symptoms at T3, relative to the sample average scores. The second class (*average-increasing*) included 19.2% of the sample and started at average levels of depression before the pandemic and increased to moderate symptoms over time. The third class (*low-stable*) represented 64.3% of the sample, who were adolescents reporting low symptoms of depression at T1 and their symptoms remained stable over time. The final trajectory class (*moderate-increasing*) represented 9.4% of the sample. Adolescents in this class reported higher-than-average levels of depressive symptoms at T1 and showed a significant increase in their symptoms over time. The four trajectories and the estimated mean changes are shown in Fig. 1. The four trajectories significantly differed in their symptoms levels at T1 [F(3,2777) = 1379.0, *p* < 0.001], at T2[F(3,2276) = 537.63, *p* < 0.001], and T3 [F(3,2091) = 2894.02, *p* < 0.001]. Depressive symptoms means and SDs for each trajectory at each time point are presented in Table 3.

**Table 3:** Mental health symptoms means and SDs for each trajectory at T1, T2 and T3.

	Anxiety symptom trajectories			Depressive symptom trajectories			
	Low-stable	Average-increasing	High-decreasing	Low-stable	Average-increasing	Moderate-increasing	High-decreasing
T1 (2019)	19.19^a^ (6.21)	24.82^b^ (6.79)	43.77^c^ (7.98)	1.84^a^ (2.03)	3.53^b^ (2.57)	6.54^c^ (3.91)	12.80^d^ (2.80)
T2 (2020)	19.60^a^ (7.48)	37.89^b^ (12.04)	39.97^c^ (12.28)	2.09^a^ (2.78)	6.27^b^ (3.85)	10.94^c^ (5.25)	8.10^d^ (5.37)
T3 (2021)	19.96^a^ (7.24)	46.14^b^ (10.66)	35.28^c^ (12.14)	1.76^a^ (1.86)	8.42^b^ (2.23)	15.27^c^ (2.39)	4.51^d^ (3.60)

Depression scores range (0–18), anxiety scores range (13–65). Different letters in the superscript indicate a significant difference between trajectories.

### Anxiety symptoms trajectories

**Figure 2:**
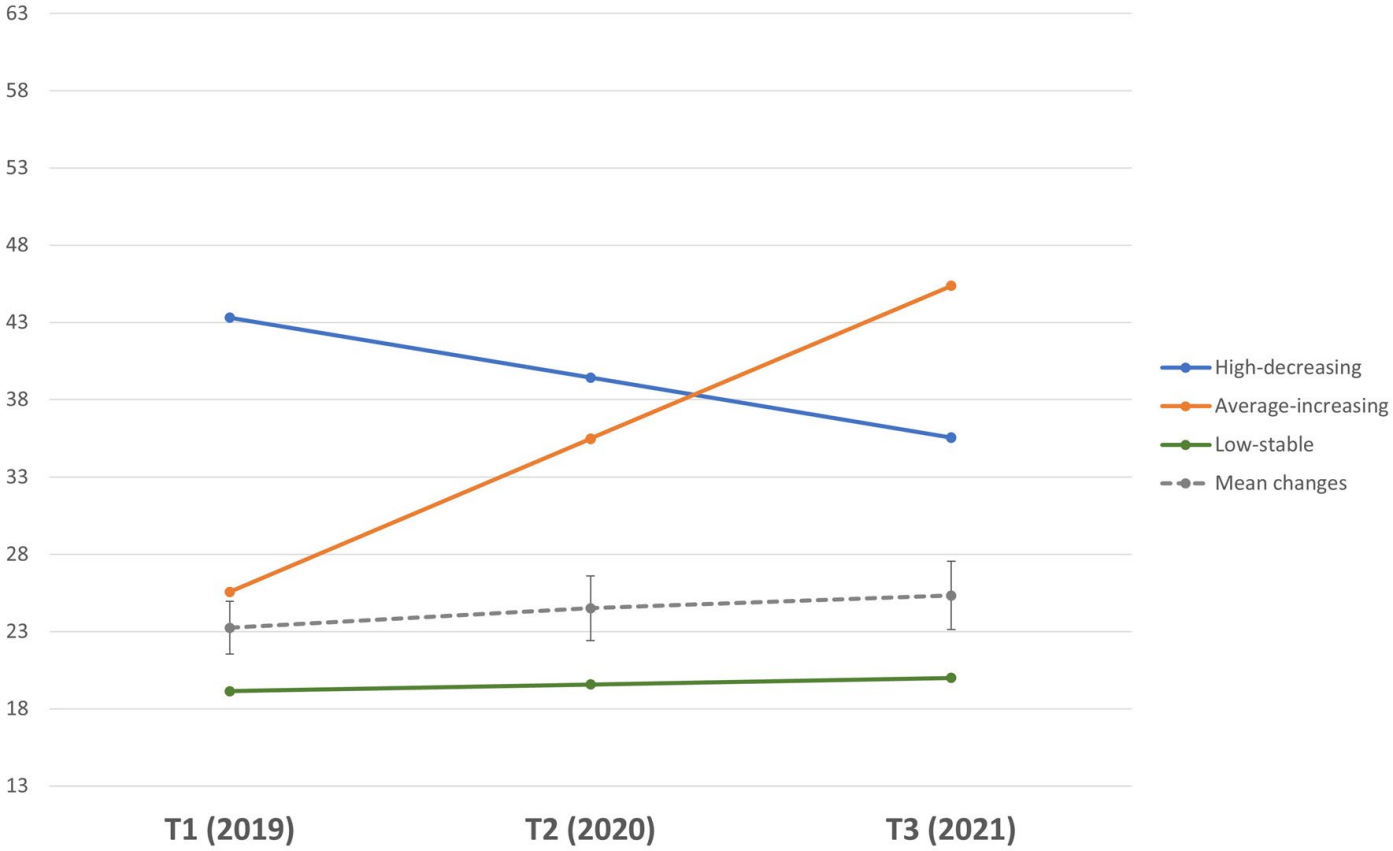
Trajectories of anxiety symptoms throughout the pandemic. *Note*. The dashed line is the average score for the whole sample at T1-T2-T3, Anxiety symptom scores ranged from 13–65.

Analysis for anxiety symptoms yielded three different trajectories (see Supplementary material). The first class (*high-decreasing*) included 13.6% of the sample. Adolescents in this trajectory showed a statistically significant decline in anxiety symptoms, but still well above the average for the whole sample. The second trajectory (*average-increasing*) included 11.6% of the sample and went from average to high symptoms from T1 to T3. The third trajectory (*low-stable*) included 74.8% of the sample and showed a slight but significant increase in anxiety symptoms over time, although still in the low range. The three trajectories and the estimated mean changes are shown in Fig. 2. The three trajectories significantly differed in their symptoms levels at T1 [F(2,2641) = 2154.50, *p* < 0.001], at T2[F(2,2182) = 979.60, *p* < 0.001], and T3 [F(2,2031) = 1281.02, *p* < 0.001]. Anxiety symptoms means and SDs for each trajectory at each time point are presented in Table 3.

### Sleep and sociodemographic characteristics among adolescents by trajectories of depressive symptoms

Sex distribution was significantly different among adolescents with different trajectories of depressive symptoms, *χ*^*2*^(6) = 151.25, *p* < 0.001. In the *high-decreasing* group, girls were the majority (58%) together with the largest proportion of adolescents answering “prefer not to say” for their sex at birth (4%). Girls were overrepresented in the *low-increasing* trajectory (67.7% vs. 30.6% boys) and in the *average-increasing* trajectory (67.8% vs. 30.7% boys). Boys represented the majority in the *low-stable* trajectory (75.7% vs. 54.5% girls). Some differences in cultural and linguistic diversity emerged, *χ*^*2*^ = 12.0, *p* = 0.007, with the *high-decreasing* trajectory having the highest proportion of adolescents with an immigrant background (18.9%), followed by the *low-stable* group (10.5%) and the *average-increasing* and *moderate-increasing* (8.3% and 8.4% respectively). Relative socioeconomic position did not differ among the trajectories *χ*^*2*^ = 3.3, *p* = 0.348.

Table 3 shows the comparisons for sleep characteristics for each of the depressive symptom trajectories. Adolescents in the *low-stable* trajectory of depressive symptoms reported significantly better sleep patterns at all time points, including the shortest SOL and WASO, the longest sleep duration, the lowest sleepiness, and the earliest chronotype. Adolescents in the *high-decreasing* trajectory of depressive symptoms showed the worst sleep patterns compared to the other groups at T1, but by T3 there were no significant differences from the low-stable group in WASO, sleep duration, and chronotype. Sleepiness and SOL were still high but no longer the highest compared to adolescents in the two increasing-symptoms trajectories. In fact, adolescents in the two increasing trajectories were better off at T1, but reported significantly longer SOL and WASO, shorter sleep duration, excessive daytime sleepiness (> 17)^30^ and later chronotype by T3. Interestingly, SOL was problematic in all trajectories, as it was close to or above the clinical guidelines of 30 min required for sleep-onset insomnia^19^.

### Sleep and sociodemographic characteristics among adolescents by trajectories of anxiety symptoms

Sex distribution was significantly different among adolescents in different trajectories of anxiety symptoms, *χ*^*2*^(4) = 181.26, *p* < 0.001. In the *high-decreasing* trajectory, girls were the largest group (73.5%) together with the largest proportion of adolescents answering “prefer not to say” for their sex at birth (2.4%). Girls were also overrepresented in the *average-increasing* trajectory (72.3% vs. 26.5% boys) whereas boys were slightly overrepresented in the *low-stable* trajectory (54.4% vs. 44.4% girls). Adolescents differed in cultural and linguistic diversity, *χ*^*2*^ = 9.11, *p* = 0.011, with the *average-increasing* trajectory having the lowest proportion of adolescents with an immigrant background (8.4%), compared to the *low-stable* (10.2%) and the *high-decreasing* group (13%). Relative socioeconomic position did not differ among the trajectories *χ*^*2*^ = 1.75, *p* = 0.416.

Table 4 shows the comparisons for sleep characteristics for each of the anxiety symptom trajectories. Adolescents in the *low-stable* trajectory of anxiety symptoms reported significantly better sleep patterns at all time points compared to the other groups, including the shortest SOL and WASO, the longest sleep duration, the lowest sleepiness, and the earliest chronotype. Adolescents in the *high-decreasing* trajectory of anxiety symptoms showed the worst sleep patterns compared to the other groups at T1, but by T3 there was no significant difference in WASO compared to the *low-stable* group and their sleep duration was no longer the shortest. Yet, sleepiness was still above recommended levels (> 17) and chronotype significantly later than the *low-stable* trajectory. Adolescents in the *average-increasing* trajectories started with similar SOL, WASO, and chronotype as the *low-stable* trajectory, but already reported a significantly shorter sleep duration and more sleepiness. By the end of the study, they were worse off in all sleep parameters. As in the depressive symptom trajectories, SOL was problematic in all groups (i.e., ≥ 30 min).

**Table 4:** Sleep characteristics among adolescents by trajectories of depressive symptoms.

	Low-stable M(SD)/%	Average-increasing M(SD)/%	Moderate-increasing M(SD)/%	High-decreasing M(SD)/%	χ^2^ (df)	*p*
SOL_sw_ T1	33.88^a^ (1.23)	45.07^b^ (2.60)	51.14^b^ (3.85)	78.06^c^ (6.74)	70.06 (3,2733)	< 0.001
SOL_sw_ T2	27.67^a^ (1.18)	42.95^b^ (2.52)	56.20^b^ (6.38)	49.22^b^ (5.55)	59.07 (3,2239)	< 0.001
SOL_sw_ T3	26.25^a^ (1.40)	47.21^bc^ (3.30)	59.07^c^ (6.66)	40.62^b^ (5.89)	56.61 (3,2053)	< 0.001
WASO_sw_ T1	14.30^a^ (0.89)	22.82^b^ (2.21)	27.37^b^ (3.21)	48.73^c^ (5.35)	66.37 (3,2733)	< 0.001
WASO_sw_ T2	8.41^a^ (0.79)	20.67^b^ (2.16)	29.00^b^ (4.21)	27.14^b^ (5.08)	62.69 (3,2239)	< 0.001
WASO_sw_ T3	7.87^a^ (0.86)	17.84^b^ (1.88)	31.69^c^ (4.39)	10.38^a^ (3.07)	48.54 (3,2053)	< 0.001
TST_sw_ T1	504.38^a^ (2.68)	477.95^b^ (5.83)	448.13^c^ (9.46)	388.32^d^ (12.54)	125.87 (3,2733)	< 0.001
TST_sw_ T2	496.50^a^ (2.76)	442.82^b^ (5.91)	410.23^c^ (12.23)	427.18^bc^ (12.61)	128.69 (3,2239)	< 0.001
TST_sw_ T3	484.17^a^ (3.01)	424.76^b^ (6.10)	360.41^c^ (14.15)	467.79^a^ (12.53)	137.04 (3,2053)	< 0.001
Sleepiness T1	12.21^a^ (0.15)	14.62^b^ (0.31)	17.85^c^ (0.49)	19.34^d^ (0.52)	309.51 (3,2733)	< 0.001
Sleepiness T2	12.11^a^ (0.17)	16.64^b^ (0.32)	20.13^c^ (0.60)	17.12^b^ (0.68)	320.93 (3,2239)	< 0.001
Sleepiness T3	12.70^a^ (0.18)	18.57^b^ (0.30)	22.32^c^ (0.59)	15.55^d^ (0.71)	456.07 (3,2053)	< 0.001
Chronotype T1	192.55^a^ (1.92)	199.55^ab^ (4.10)	218.82^bc^ (6.58)	226.79^c^ (9.62)	28.86 (3,2733)	< 0.001
Chronotype T2	209.39^a^ (2.15)	233.45^b^ (4.62)	255.68^c^ (8.99)	239.69^bc^ (9.47)	52.14 (3,2239)	< 0.001
Chronotype T3	227.51^a^ (2.47)	262.80^b^ (4.71)	286.90^c^ (10.09)	233.24^a^ (9.14)	68.15 (3,2053)	< 0.001
Sleep-in_we_ T1	85.15^a^ (2.20)	85.87^a^ (4.47)	110.10^b^ (6.48)	101.06^ab^ (9.73)	16.05 (3,2733)	= 0.001
Sleep-in_we_ T2	85.44^a^ (2.31)	102.53^b^ (4.63)	109.92^b^ (9.36)	109.82^b^ (8.99)	20.91 (3,2239)	< 0.001
Sleep-in_we_ T3	99.04^a^ (2.61)	119.61^b^ (4.53)	118.42^ab^ (11.53)	109.85^ab^ (10.62)	15.51 (3,2053)	= 0.001

*SOL* sleep onset latency, *WASO* wake after sleep onset, *TST* total sleep time, Chronotype = Midpoint of Sleep on Free days (corrected for weekday sleep), Sleep-in_we_ = Weekend wake-up time–Weekday wake-up time. Sleepiness ranged from 0 to 32, the cutoff for excessive sleepiness is 14 for boys and 17 for girls^28^. Different letters in the superscript indicate a significant difference between trajectories.

## Discussion

The aim of this study was to explore whether healthy sleep patterns were protective of adolescent mental health throughout the COVID-19 pandemic.

In the present study, both depression and anxiety symptoms generally increased over time. While this trend is similar to pre-pandemic studies for depression, it is in the opposite direction for anxiety, whose symptoms have previously shown to decline from childhood throughout adolescence^31^. A recent meta-analysis of mental health changes throughout the pandemic shows a clear increase in depressive symptoms in children and adolescents, and a slight increase in anxiety symptoms, with girls generally showing worse mental health^6^. Similar to previous trajectory-studies examining depressive and anxiety symptoms^32,33^, in the present study, not all adolescents displayed increasing symptoms: the majority of adolescents reported stable-low symptoms of both anxiety and depression, and a small portion of adolescents showed improving mental health. Identifying modifiable protective factors reported by adolescents in these low and declining symptom trajectories is crucial for promoting mental health in this population. In this study, we examined sleep as a potential protective factor and found that adolescents in low-risk trajectories of anxiety and depression consistently reported the healthiest sleep patterns. These healthy sleep characteristics included: (1) longer weekly sleep duration, (2) earlier chronotype, (3) lower sleepiness, (4) shorter sleep latency and time awake during the night. Weekend catch-up was significantly shorter for the *low-stable* trajectory of depression but was not significantly different between the trajectories of anxiety. When looking at the divergent trajectories of increasing vs decreasing symptoms of anxiety and depression, some interesting differences emerged.

Among adolescents whose depressive symptoms improved over time, sleep duration no longer differed from the low-risk trajectory by the end of the study. Similarly, for adolescents whose anxiety symptoms improved over time, their sleep duration was no longer the shortest compared to increasing-symptom trajectories. This is not surprising, as studies examining the link between sleep duration and mental health have found a dose-response association that supports the positive association between the recommended 8–10 h of sleep for adolescents and emotional health^34,35^. Chronotype followed similar trends as sleep duration, as a later chronotype leads to later bedtimes during schooldays, shortening the sleep opportunity when wake-times are fixed^36^. In contrast, an early chronotype seems to be protective for adolescents’ mental health, in line with previous studies during the pandemic^26,27^. An interesting finding was that the chronotype of adolescents in the high-decreasing trajectory of depression was significantly later than the other trajectories at T1, but did not differ from adolescents in the low-risk trajectory by the end of the study. Conversely, chronotype became significantly later for adolescents in the two increasing trajectories of depressive symptoms. This suggests that the natural delay in sleep timing slowed for teenagers whose depressive symptoms improved, while it delayed further for adolescents with increasing symptoms. This finding is worth exploring further, as an earlier chronotype has been found to be protective for depression for several reasons, not only because it is linked to earlier bedtimes and longer sleep duration, but also because it better aligns with the environment (e.g., bright light exposure) and with social obligations (e.g., morning school times)^18^. One hypothesis may be that for some adolescents, the more unstructured nature of online classes could have prompted more irregular sleep routines, making room for a delay in bed- and wake-times (and a later sleep midpoint/chronotype). However, some adolescents were able to increase their sleep duration and maintain (rather than delay) their timing. In this study, we did not directly assess lockdown status, but a previous study found a general increase in sleep duration in this population, independent of lockdown measures^25^. This might also explain the relatively small difference in weekend catchup between trajectories. Future studies should further explore what may explain these variations in chronotype, including individual characteristics and contextual factors (e.g., self-control, parental rules about sleep).

In addition to sleep quantity, perceived sleep quality is an important aspect closely linked to mental wellbeing^22^. Experts agree that measures of sleep continuity are good indicators of sleep quality^19^. In particular, time taken to fall asleep as well as time awake at night are hypothesised as important mechanisms for both anxiety and depression, as they create an opportunity for worry at a time when more adaptive emotion regulation strategies might not be activated^12^. Although SOL was markedly long among all adolescents (> 30 min), it was consistently shorter in the low-risk trajectories of both anxiety and depression, and no longer the longest among decreasing-symptom trajectories. Time awake at night followed similar trends. Finally, we also examined daytime complaints in relation to depression and anxiety symptoms. In the present study, teenagers who belonged to improving mental health symptom trajectories no longer reported the highest sleepiness by the end of the study, compared to worsening mental health symptom trajectories. Feelings of tiredness have been previously found to be both cross-sectionally and longitudinally linked to anxiety and depression in adolescents^22^. All in all, these results show significantly better sleep quality and quantity and less daytime sleepiness among adolescents with better mental health across the pandemic years. Furthermore, the divergent trajectories of anxiety and depressive symptoms clearly show that sleep patterns followed closely.

Sleep and mental health are closely intertwined. The aim of this study was not to discern directionality, rather we aimed to use a person-oriented perspective to inform *who* is at risk for developing problems, and poor sleep characteristics were a common determinant among risk-trajectories for both anxiety and depressive symptoms. Some differences in sleep patterns existed from the start of the study—for example, adolescents in the average-increasing trajectory of depression already reported overall worse sleep health compared to the low-stable trajectory. For the average-increasing trajectory of anxiety, sleep duration and sleepiness were distinct from the low-risk adolescents. These differences in sleep patterns could help explain why the pandemic sparked an increase in mental health problems in these adolescents and can potentially aid in the identification of individuals in need of early interventions. In fact, a recent study found that stressful events were followed by an increase in sleep problems and later increases in anxiety symptoms within a few months among adolescents^37^. Nonetheless, it might still be difficult to identify these emerging risk-groups (average-increasing trajectories), as their sleep was significantly different from the low-stable trajectories but it was not yet problematic (e.g., sleep duration close to the recommended 8 h/night^20^; see Tables 4, 5). This supports the importance of promoting sleep health in the adolescent population in a universal manner, as some of the adolescents at-risk might easily be missed. Given that the link between sleep and mental health is ultimately bi-directional and can create a vicious cycle of poor emotional regulation and poor sleep quality and quantity^38^, breaking this cycle by targeting sleep is warranted and has shown promising benefits for mental health^9^. For example, sleep has shown significant improvements (sleep onset latency in particular) alongside symptoms of depression following bright light therapy, cognitive behavioural therapy for insomnia (CBT-I), school-based sleep interventions^9^, and later school-start times^39^. Two recent meta-analyses found evidence that improving sleep can lead to an improvement in both anxiety and depressive symptoms^40,41^. In addition, addressing sleep problems first can be advantageous because it is less stigmatized and easier to talk about compared to mental health^40^.

**Table 5:** Sleep characteristics among adolescents by trajectories of anxiety symptoms.

	Low-stable M(SD)/%	Average-increasing M(SD)/%	High decreasing M(SD)/%	χ^2^ (df)	*p*
SOL_sw_ T1	35.55^a^ (1.07)	43.01^a^ (3.54)	68.61^b^ (4.22)	63.41 (3,2733)	< 0.001
SOL_sw_ T2	29.35^a^ (1.05)	53.97^b^ (4.79)	47.50^b^ (3.69)	50.18 (3,2226)	< 0.001
SOL_sw_ T3	29.81^a^ (1.30)	54.61^b^ (4.55)	44.67^b^ (4.41)	37.68 (3,2047)	< 0.001
WASO_sw_ T1	15.29^a^ (0.79)	19.67^a^ (2.56)	43.91^b^ (3.45)	69.91 (3,2733)	< 0.001
WASO_sw_ T2	9.71^a^ (0.73)	30.90^b^ (3.54)	22.36^b^ (2.95)	54.26 (3,2226)	< 0.001
WASO_sw_ T3	8.74^a^ (0.75)	27.62^b^ (3.19)	16.91^a^ (2.62)	43.05 (3,2047)	< 0.001
TST_sw_ T1	499.79^a^ (2.38)	479.75^b^ (7.86)	412.89^c^ (8.32)	108.52 (3,2733)	< 0.001
TST_sw_ T2	490.14^a^ (2.44)	410.92^b^ (9.90)	431.85^b^ (7.89)	113.88 (3,2226)	< 0.001
TST_sw_ T3	475.00^a^ (2.66)	385.36^b^ (10.22)	441.40^c^ (8.62)	84.88 (3,2047)	< 0.001
Sleepiness T1	12.77^a^ (0.14)	14.81^b^ (0.42)	18.24^c^ (0.38)	203.93 (3,2733)	< 0.001
Sleepiness T2	12.77^a^ (0.15)	18.17^b^ (0.47)	18.21^b^ (0.44)	256.61 (3,2226)	< 0.001
Sleepiness T3	13.71^a^ (0.16)	20.12^b^ (0.43)	18.27^c^ (0.46)	274.02 (3,2047)	< 0.001
Chronotype T1	196.05^a^ (1.72)	198.05^ab^ (5.47)	214.18^b^ (5.90)	8.92 (3,2733)	= 0.01
Chronotype T2	214.29^a^ (1.91)	242.17^b^ (6.98)	236.33^b^ (6.18)	26.85 (3,2226)	< 0.001
Chronotype T3	235.66^a^ (2.14)	264.92^b^ (6.96)	251.64^b^ (6.91)	20.58 (3,2047)	< 0.001
Sleep-in_we_ T1	86.16^a^ (1.93)	93.61^a^ (5.97)	98.34^a^ (6.12)	5.11 (3,2781)	= 0.078
Sleep-in_we_ T2	88.85^a^ (2.02)	109.41^b^ (7.31)	100.98^ab^ (6.18)	10.87 (3,2226)	= 0.004
Sleep-in_we_ T3	104.43^a^ (2.22)	116.28^a^ (7.89)	110.27^a^ (7.05)	2.68 (3,2047)	= 0.261

*SOL* sleep onset latency, *WASO* wake after sleep onset, *TST* total sleep time, Chronotype = Midpoint of Sleep on Free days (corrected for weekday sleep), Sleep-in_we_ = Weekend wake-up time–weekday wake-up time. Sleepiness ranged from 0 to 32, the cutoff for excessive sleepiness is 14 for boys and 17 for girls^28^. Different letters in the superscript indicate a significant difference between trajectories.

The present study has a number of strengths and limitations that need to be taken into account when interpreting the results. Mental health symptoms and sleep patterns were self-reported, which can be subject to error and common method bias. However, externally developed measures that have been validated in adolescents were used. Although objective sleep measures are encouraged in future studies, self-reported sleep has proven valid compared to actigraphy^42,43^. This is also a more feasible method when including a large sample of adolescents followed over time. The large and diverse sample, spanning three Australian states is a strength of the study, together with the longitudinal design, which enabled to capture changes throughout the pandemic. Despite the three waves of longitudinal data, the results of this study cannot discern whether sleep patterns or mental health precede one another. More frequent longitudinal sampling could have provided a clearer picture^44^. However, using sophisticated person-oriented analyses allowed to highlight that not all adolescents were impacted the same way during the COVID-19 pandemic. In fact, the majority of the sample coped well during this stressful time (low-stable trajectories) and these low-risk classes also reported the healthiest sleep patterns. In addition, although WASO and SOL decreased over time in the whole sample, when looking at subgroups the decrease occurred only among adolescents whose symptoms improved. These important results might have been missed if only average changes were examined.

To conclude, sleep and mental health go hand-in-hand for adolescents. During a stressful time such as the COVID-19 pandemic, good sleep health was distinctive of adolescents who maintained low and stable symptoms of anxiety and depression. Given the bidirectional nature of the link between sleep and mental health, promoting healthy sleep habits in adolescents is a promising modifiable factor to improve and maintain mental health. Adolescent mental health is a public health priority^8^, and this study provides empirical evidence that sleep health should be one central target for prevention and intervention.

## Method

### Design and participants

This study utilises data from the “Health4Life” cluster randomised controlled trial, which aimed to evaluate the efficacy of an eHealth intervention targeting six modifiable risk factors among Australian adolescents (sleep, physical activity, diet, screen time, alcohol use and tobacco smoking). Baseline data were collected in 2019 (approximately July-November) using online self-report surveys among Year 7 students at 71 secondary schools across New South Wales (NSW), Queensland (QLD) and Western Australia (WA), with follow up surveys conducted in 2020 (approximately July-December) and 2021 (approximately July-December). To avoid contamination effects from the intervention, this study focuses on control group data. Only students with data on depressive and anxiety symptoms at baseline were included (Depressive symptom trajectories: N_T1_ = 2781, N_T2_ = 2280, N_T3_ = 2095; Anxiety symptom trajectories: N_T1_ = 2781, N_T2_ = 2267, N_T3_ = 2088). Participants provided written consent and parents provided passive, active written or active verbal consent, depending on the approved procedures for the school/region. The Health4Life trial was performed in accordance with relevant guidelines and regulations, it was registered with the Australian and New Zealand Clinical Trials registry (ACTRN12619000431123) and has ethical approval from ten relevant committees (University of Sydney HREC2018/882, NSW Department of Education SERAP 2019006, University of Queensland 2019000037, Curtin University HRE2019-0083 and several Catholic Diocese committees). The study protocol provides further details on recruitment and consent procedures^29^.

### Measures

#### Sociodemographic characteristics

Sociodemographic factors included sex assigned at birth, age, cultural and linguistic diversity (CALD), and relative socioeconomic position. CALD was defined as per recommendations from a recent Australian review^45^ to include participants who were born in a non-English speaking country and/or primarily speak a language other than English at home. Relative family affluence was identified using the Family Affluence Scale III (FASIII), which has demonstrated good test–retest reliability (r = 0.90) and strong correlation with parental report^46^. The FASIII generates a summed score across indicators of familial wealth (e.g., number of computers, number of bathrooms in home, etc.) as a proxy for familial socioeconomic status that children and adolescents might be better at reporting compared to parent or caregivers’ income and education.

#### Sleep patterns

Average sleep duration per night was measured using the validated Modified Sleep Habits Survey^42,43^. Students reported the time they usually: (1) went to bed (time—12 h format), (2) attempted sleep (time—12 h format), (3) took to fall asleep (duration—h, min), (4) were awake during the night (duration—h, min), and 5() woke up in the morning (time—12 h format) over the past week (separately for week nights and weekend nights). In the present study, we derived several sleep parameters, including sleep onset latency (SOL; i.e., time taken to fall asleep), wake after sleep onset (WASO; i.e., time awake during the night), total sleep time (TST; i.e., time between falling asleep and wake up in the morning, minus WASO) during schooldays, and chronotype. We calculated chronotype as the midpoint of sleep on free days (i.e., sleep onset time on weekends plus sleep duration on weekends, divided by 2). According to Roenneberg et al.^47^ a correction needs to be made as many adolescents restrict their sleep on schooldays and attempt to catch up on weekends. Therefore, if the sleep duration on weekends is longer than on schooldays, sleep midpoint is calculated as sleep onset time (weekends) plus sleep duration (average schoolday-weekend) divided by 2. The midpoint of sleep is a good behavioural marker for circadian phase^47^. Finally, to examine irregular sleep patterns during weekdays and weekends, we calculated “weekend sleep-in” by subtracting wake-up time on weekends—wake-up time on weekdays^48^.

#### Sleepiness

The Pediatric Daytime Sleepiness Scale was used to assess daytime sleepiness^30^. It has been validated in adolescents and includes eight items such as ‘*How often do you fall asleep or feel drowsy in class?*’ and ‘*How often do you have trouble getting out of bed in the morning?*’ with five response options from ‘*Never*’ (0) to ‘*Very often/ always*’ (4), with scores ranging from 0 to 32. The cutoff for excessive sleepiness is 14 for boys and 17 for girls^30^. The Cronbach’s alpha was acceptable (α = 0.72).

#### Anxiety

Past 7-day anxiety symptoms were assessed with the PROMIS Anxiety Paediatric (PROMIS-AP) scale, which has been validated among adolescents^49^. The 13-item scale asks participants to report frequency of symptoms including difficulty relaxing, and feelings of nervousness, worry, and fear, amongst others on a scale from *‘never’* (1) to ‘*almost always’* (5), the total ranging from 13–65^49^. The Cronbach’s alpha was 0.94.

#### Depression

Past 7-day depressive symptoms were measured using the modified Patient Health Questionnaire for Adolescents scale (PHQ-A)^50^. The 9-item scale asks participants to report how often they experienced symptoms such as “feeling down, depressed, irritable or hopeless”, sleep issues, tiredness, and changes in appetite, weight or behaviours on a scale from *‘not at all’* (0) to *‘nearly every day’* (3)^50^. The 9^th^ item (measuring thoughts of death and self-harm) was removed on request of the ethics board. In addition, 2 items were excluded from analyses because of their overlap with sleep, our variable of interest. Depression symptoms were analysed using a sum of the remaining six items, ranging from 0–18 (Cronbach’s alpha = 0.84).

### Data analysis

We followed a two-step procedure to identify different mental health trajectories^51^. In Step 1, we estimated a latent curve growth model to examine how, on average, adolescents’ mental health symptoms changed over a 2 years period, and to examine whether there was a significant between-person variation in intercept (initial level) and slope (change over time) of depression and anxiety. In Step 2, we used latent class growth analysis (LCGA) to identify adolescents with different mental health trajectories. To determine the number of trajectories that best fit the data we looked for the lowest Bayesian Information Criterion (BIC), highest entropy (i.e., classification accuracy), non-significant Lo–Mendell–Rubin test (LMR; i.e., model fit improvement from *n* − 1 to *n* classes), and the proportion of adolescents in each group trajectory (> 5%)^52^. When these indicators were contrasting, we chose the most parsimonious solution (i.e., fewer classes)^52^. We analyzed trajectories for depressive symptoms and anxiety separately. To validate the distinctiveness of the classes, we compared depression and anxiety symptom trajectories on symptom levels at each time point using ANOVAs. After establishing the different trajectories, we compared adolescents in each trajectory on sex distribution and sleep parameters across three time points using the BCH method in Mplus^53^. This method uses a weighted multiple group analysis, where the groups correspond to the latent classes, and therefore the classes established in the LCGA model are not affected in this second step (i.e., when analysing differences in sleep patterns)^53^. Data were analysed in SPSS (v. 26) and Mplus^54^. We handled missing data in Mplus using full information maximum likelihood (FIML). Depressive symptoms model: 4 missing data patterns, covariance coverage ranged between 0.67 and 1.00. Anxiety model: 4 missing data patterns, covariance coverage ranged between 0.67 and 1.00. The covariance coverage was well above the recommended 0.10 to reliably use FIML. Moreover, FIML is a superior method compared to mean imputation, listwise deletion or pairwise deletion, as it provides more reliable standard errors^55^.

### Supplementary Information

Supplementary Information.

## Data Availability

The data underlying this article cannot be shared publicly due to the agreement made with the individuals that participated in the study. The data will be shared on reasonable request to the corresponding author.
