# Supplementary material for: Adolescents’ trajectories of depression and anxiety symptoms prior to and during the COVID-19 pandemic and their association with healthy sleep patterns
Source: Sci Rep. 2024 May 10;14:10764. doi: 10.1038/s41598-024-60974-y (PMC11087504; doi:10.1038/s41598-024-60974-y)
Supplement: Supplementary file 1 — Supplementary Information. [file 41598_2024_60974_MOESM1_ESM.docx]

Adolescents’ trajectories of depression and anxiety symptoms prior to and during the COVID-19 pandemic and their association with healthy sleep patterns

Serena Bauducco ^a, b^

Lauren A. Gardner^c c^

Scarlett Smout ^c^

Katrina E. Champion^c^

Cath Chapman^c^

Amanda Gamble ^d^

Maree Teesson ^c^

Michael Gradisar ^e,f^

Nicola C. Newton ^c^

^a^ Flinders University, Adelaide, Australia.

^b^ Örebro university, Örebro, Sweden.

^c^ The Matilda Centre for Research in Mental Health & Substance Use, The University of Sydney, Australia.

^d^ The Woolcock Institute, The University of Sydney, Sydney, Australia.

^e^ WINK Sleep Pty Ltd, Adelaide, Australia.

^f^ Sleep Cycle AB, Gothenburg, Sweden.

## Supplementary material

Latent class growth models (LCGMs) results:

As shown in Table 1s, for depressive symptoms, BIC improved from one-class to a five-class solution. The LMR-LRT test (Lo-Mendell-Rubin Likelihood Ratio Test) [51] was not significant for the 5-class solution (*p* = .06), indicating that there was no significant improvement in model fit to the data from a 4-class to a 5-class solution. Although the entropy was better for the 2- and 5-class solution, the 4-class solution was retained because it represents a good balance between parsimony and complexity.

For anxiety symptoms, the class solutions fit indices BIC (Bayesian Information Criterion) and entropy improved from the one-class to a three-class solution [51]. The LMR-LRT test was not significant for the 4-class solution, suggesting that the 3-class solution fits the data better.

| **Table 1s: Model fit statistics for analysis of growth trajectories of depression and anxiety over two years.** | | | | | | |
| --- | --- | --- | --- | --- | --- | --- |
| **Depression** | | | | | | |
|  | **Entropy** | **AIC** | **BIC** | **Adj. BIC** | **Adj. LRT** | **p** |
| 2 cluster | 0.783 | 39931.64 | 39979.08 | 39953.66 | 1347.85 | <.001 |
| 3 cluster | 0.834 | 39235.66 | 39300.9 | 39265.95 | 669.26 | <.001 |
| **4 cluster** | 0.789 | 39007.89 | 39090.92 | 39046.44 | 224.34 | 0.01 |
| 5 cluster | 0.805 | 38717.83 | 38818.65 | 38764.64 | 264.98 | 0.06 |
| 6 cluster | 0.8 | 38551.18 | 38669.79 | 38606.24 | 175.97 | 0.418 |
| 7 cluster |  |  |  |  |  |  |
| **Anxiety** | | | | | | |
|  | **Entropy** | **AIC** | **BIC** | **Adj. BIC** | **Adj. LRT** | **p** |
| 2 cluster | 0.824 | 53545.95 | 53593.40 | 53567.98 | 1759.47 | <.001 |
| **3 cluster** | 0.815 | 53139.07 | 53204.30 | 53169.35 | 396.23 | <.001 |
| 4 cluster | 0.825 | 52804.45 | 52887.48 | 52842.99 | 326.88 | 0.09 |
| 5 cluster | 0.648 | 52810.45 | 52911.26 | 52857.25 | 0 | 0.352 |
|  |  |  |  |  |  |  |
